# Gender differences in walking (for leisure, transport and in total) across adult life: a systematic review

**DOI:** 10.1186/s12889-017-4253-4

**Published:** 2017-04-20

**Authors:** Tessa M. Pollard, Janelle M. Wagnild

**Affiliations:** Department of Anthropology, Durham University, Dawson Building, South Road, Durham, DH1 3LE UK

**Keywords:** Walking, Gender, Men, Women, Leisure, Transport, Travel, Aging

## Abstract

**Background:**

The aim of this systematic review was to examine gender differences in walking for leisure, transport and in total in adults living in high-income countries, and to assess whether gender differences in walking practices change across the life-course.

**Methods:**

A systematic literature search was conducted of publications dated 1995 to 2015. Papers providing quantitative data on participation in walking of both men and women aged at least 18 years in a high-income country were screened for the quality of the data on gender differences in walking. Data were extracted and results were synthesised using forest plots and narrative summary.

**Results:**

Thirty-six studies were included in the review: 18 reported on walking for leisure, 16 on walking for transport (in total, or for particular purposes), and 14 on total walking. Most (33) studies provided data comparing the proportion of men and women who walked (at all or for a minimum duration) over a defined period, usually one week. There was consistent evidence that more women than men walk for leisure, although effect sizes were small. However, this effect varies by age: more younger women than younger men walk for leisure, but the gender difference diminishes with age and appears to reverse in the oldest age groups. Taking all ages together, there was no consistent gender difference in walking for transport or in total walking, although the small number of studies reporting on walking to undertake errands suggested that more women than men walk for this purpose.

**Conclusions:**

While there is little evidence that levels of total walking consistently vary by gender, our findings suggest that there are consistent gender differences in participation in walking for some purposes, including for leisure, and that there are gender differences in the impact of age on walking. We conclude that more research is needed to improve our understanding of how walking fits into the lives of women and men across the life-course, especially in relation to gender differences in the impact of aging on walking.

**Prospero registration:**

PROSPERO registration number: CRD42015025961.

**Electronic supplementary material:**

The online version of this article (doi:10.1186/s12889-017-4253-4) contains supplementary material, which is available to authorized users.

## Background

Walking is associated with better mental and physical health and reduced mortality [1, 2] and, when used for transport, with reduced air and noise pollution [3]. In contrast to other forms of physical activity, walking has the advantage of being accessible to most people. For these reasons, promotion of walking has become more prominent in public health campaigns [4–6]. With rare exceptions [7, 8], the possibility of gender differences in walking practices has largely been ignored, but an understanding of whether, how and why walking practices differ between men and women would help inform such campaigns.

Within the widely used ecological model of health behaviour, gender is usually identified as a personal characteristic that may modify, at the individual level, the impact of wider social and environmental influences on behaviours such as walking [9, 10]. However, gender is social construct with greater power than this [8, 11]. Gender codes the body, structures social relations (e.g. the division of labour and responsibilities) and affects access to resources [12]. Transport and exercise related activities, technologies such as the car, and environments such as ‘the street’ also have gendered meanings [12]. As a consequence, daily mobility is powerfully shaped by gender [12], as exemplified in the consistent finding in countries such as the USA and Germany that women make, on average, shorter trips than men (partly because they tend to work closer to home than men [13, 14]). Gender differences in the types of physical activity undertaken for leisure are also widely recognised, for example more men than women participate in team sports in the USA and across the European Union [15, 16]. We predict that walking, which is undertaken both for transport and for leisure, will also be gendered.

The aim of this systematic review is to assess the current evidence on gender differences in walking in high income countries. Because gender is routinely used to organise data presentation, and is often included in multivariate models in studies examining other (usually environmental) determinants of walking, data on gender differences in walking are available. We hypothesised that there are gender differences in participation in walking for leisure, for transport, and in total walking. We also set out to examine whether gender differences change across the life-course. Both walking practices and the impact of gender on everyday life vary across societies and we restricted the review to high income countries to reduce such variation, while remaining alert to the likely relevance of geographical variation in gender differences.

## Methods

The protocol for this review is registered at https:www.crd.york.ac.uk/PROSPERO (registration number CRD42015025961).

### Eligibility criteria

Studies were eligible if they met the following inclusion criteria: 1) provides quantitative data on both men’s and women’s walking in everyday life in a form allowing assessment of effect size; 2) participants at least 18 years old; 3) published 1995-2015 reporting data collected in 1990 or later (to limit variation due to changes in gender roles and walking practices over time); 4) written in English 5) study setting must be a high-income country as defined by the World Bank (http://www.data.worldbank.org/about/country-and-lending-groups#High_income). Exclusion criteria were: 1) studies including only people with certain characteristics, e.g. particular ethnic groups, employed people, patients (other than groups defined by age or ability to walk); 2) inclusion only of people who engage in walking or in particular types of walking; 3) intervention-based studies unless pre-intervention data were available. As we were interested only in ‘walks’ rather than total number of steps taken, we did not include studies that assessed only number of steps.

### Information sources and search strategy

A search of the literature was conducted in February 2016 using Web of Science Core Collections, PubMed, and the Transportation Research International Documentation (TRID) databases. The search strategy included a combination of terms for walking (walk, walking, pedestrian*, active travel) and adults (men AND women, gender*, adult, adults), with exclusion terms for certain types of studies (e.g., clinical trials, reviews). The grey literature (http://www.opengrey.eu, http://www.greylit.org), the reference lists of all included papers, and the archives of the *Journal of Physical Activity and Health* and the *International Journal of Behavioral Nutrition and Physical Activity* were also searched.

### Study selection

Study selection occurred in two phases. First, titles and abstracts of studies were screened for relevance. This task was shared between the authors, and agreement between authors was checked in an initial sample of 50 articles to ensure consistency. Articles with relevant abstracts were divided between the authors and the full text was consulted to determine eligibility. As with abstracts, agreement between authors was checked at the beginning. All full texts deemed eligible by one author were confirmed by the other author. In cases of disagreement between authors, the differences were discussed until a joint decision was made. Where more than one paper used the same dataset or survey to report on the same type of walking (leisure, travel or total), a joint decision about which paper to include in the review was made, with preference given to data from more recent survey years.

### Quality assessment

The quality of each study was assessed taking the Critical Appraisal Skills Programme (CASP) as a starting point. We assessed the quality of the study design including recruitment strategy, the use of appropriate methodology to assess walking, correct use of statistical models where relevant, and the clarity and completeness of reporting of findings. This involved assessing risk of bias relevant to gender having been introduced in the recruitment strategy or in assessment of walking. We applied these criteria specifically to data on gender differences in walking. Some studies used methods that were appropriate for their research aims but did not provide good quality data on gender differences and were therefore excluded. The quality of each study was assessed independently and confirmed by both researchers. Discrepant assessments were discussed until agreement was reached.

### Data extraction

For all articles deemed eligible for inclusion in the review, we extracted data on location, date of data collection, methodology, characteristics of sample, and results for gender differences in leisure, transportation, or total walking. Where participation in walking was reported in more than one way in a paper, we extracted results for the variable closest to the prevalence of any walking in a week. Data were extracted independently by one author and checked for each paper by the other author.

### Synthesis of results

The findings of this review are presented as a numerical and textual summary, rather than through meta-analysis, due to the wide variation in methodology and measures in the included studies, and also because gender roles may vary across societies.

For prevalence data, odds ratios and 95% confidence intervals comparing the proportion of women who walked with the proportion of men were taken from the paper or, if necessary, calculated. An odds ratio of above 1 indicates that more women walked than men and below 1 that fewer women walked than men. Unadjusted odds ratios were preferred; if only adjusted odds ratios were given in the original papers and it was possible to calculate unadjusted odds ratios, we did so. Forest plots were constructed including studies that reported the prevalence of up to 30 min of walking over a specified time period. Logarithmic odds ratios were plotted on a linear scale so that effect sizes were visually equivalent above and below 1. Where papers reported data separately for different age groups, we plotted these odds ratios in a separate plot. Odds ratios for gender differences not included in the forest plots are given in the text in the form OR (95% confidence intervals). The results of tests for gender differences for other outcomes are also given in the text.

In the synthesis, studies are cited using the first author’s name for identification and giving the location of the study and age range of participants, to facilitate interpretation.

## Results

### Study selection

**Fig. 1:**
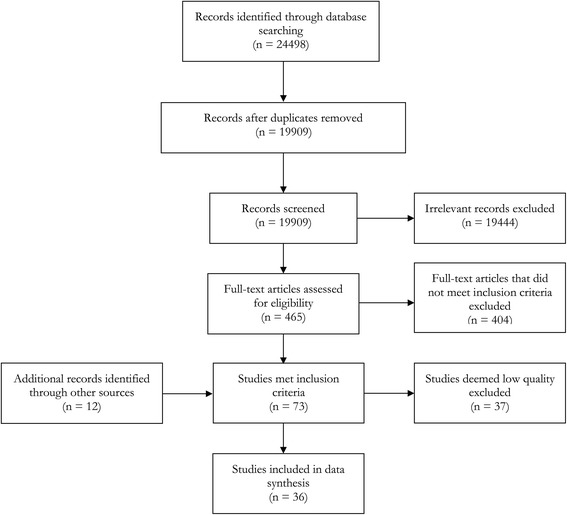
Flow chart of the study selection process

The results of the database searches are summarised in Fig. 1 and led to 36 studies being included in the analysis [17–52]. Just over half of the eligible studies were excluded because the information they provided on gender differences in walking was deemed to be of low quality for a variety of reasons, including lack of clarity or omission in reporting study details, inadequate measures of walking or inappropriate use of parametric statistical methods. We did not identify risk of bias specific to particular studies, but all included studies assessed walking by self-report and it is possible that the self-reports of men and women in relation to walking are subject to different biases. Different outcomes (leisure, transport or total walking, age groups combined versus separate age groups) of the same study or survey were reported in different included papers in two cases [20, 32, 40, 50, 51].

### Study characteristics

Fourteen studies were from North America, eleven from Europe, five from Australia and four from Asia (Additional file 1). Two papers analysed combined data from North America, Australia and Europe. The oldest reported date of data collection was 1996 [22] and the most recent 2014 [34]. Sample sizes varied from 474 to 199,400 (Additional file 1). While most studies reported a response rate, some did not, and different studies used different methods for assessing their response rate, so that it was impossible to compare response rates across studies.

### Measurement of walking

All the studies assessed walking by self-report. Most studies either used the validated International Physical Activity Questionnaire (IPAQ) (which asks respondents to report how many days over the last 7 days they walked for at least 10 min and then to report how much time they usually walked on days when they walked) [21, 26, 34, 43, 46, 48, 50, 51] or employed a very similar approach combining frequency and duration of walking [18–20, 23, 24, 27–29, 31, 32, 35–38, 40–42, 44, 45, 52]. Others asked only about the frequency of walking [17, 25, 30, 33, 49] or the duration of walking [22, 39] over a particular period, usually one week. One study derived information on walking from data on time-use for a period of 24 h [47].

Most (22) studies derived a walking variable that assessed the prevalence of doing any or at least 10 min walking over a particular period (usually one week) (Additional file 1). Others (8) looked at the prevalence of walking for a longer minimum duration (most often 150 min) over a particular period. Three studies assessed whether participants walked for at least 30 min on at least 5 days per week, or for 150 min in total and at least 5 times per week, a measure we call ‘regular substantial walking’. Two studies examined the duration of walking over a week and one study examined the number of walks taken over the past 7 days.

Eighteen papers reported on walking for leisure, 16 papers reported on walking for transport and 14 papers reported on total walking (Additional file 1).

### Results of studies and synthesis of findings

#### Gender differences in walking for leisure

##### Walking for leisure (general)

**Fig. 2:**
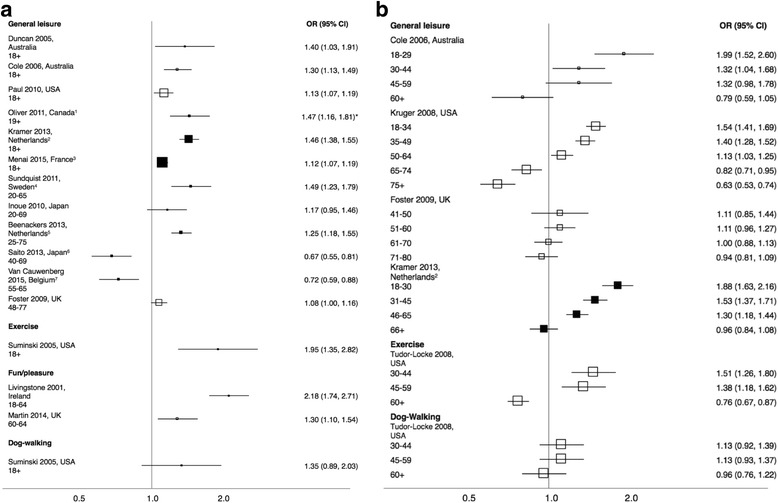
Forest plot showing odds ratios for the prevalence of walking for leisure amongst women in comparison to men among (**a**) all participants and (**b**) by age group. Clear markers indicate bivariate analysis; filled markers indicate multivariate analysis, and footnotes detail control variables included in each model. Marker size is proportional to the sample size of the study. * Results plotted here from one of several models in original study; reported ORs (95% CI) ranged from 1.47 to 1.49 (1.16 to 1.19, 1.81 to 1.85). ^1^ Age, income, marital status, chronic conditions, obesity, neighbourhood income, land use. ^2^ Age, ethnicity, household composition, education, household income and wealth, neighborhood population density. ^3^ Age, income, working status, self-rated health status, smoking status, leisure screen time, city density, population. ^4^ Age, family income, marital status, neighborhood SES and walkability. ^5^ Age, gender, education, country of origin. ^6^ Age, education, employment, household economic status, presence of children in household, self-rated health, social environment, aesthetics, and presence of pain due to orthopedic disorders. ^7^ Age, suburb SES, other recreational MVPA, education, retirement status, functional limitations, urban vs rural, park proximity and quality

There is clear evidence that more women than men walk for leisure when all age groups are considered together, although the effect size is small (Fig. 2a). Odds ratios by age group (Fig. 2a and b) suggest that at younger ages more women walk for leisure than men but that this gender difference diminishes progressively with age, with evidence that it reverses in the oldest age groups so that more older men than older women walk for leisure. Results were consistent across countries.

Van Dyck (International, 20-65) [51] found no significant gender difference in weekly minutes of walking for leisure (OR 1.02 (0.94, 1.10)) in models adjusting for age, marital status, education, body mass index, area-level household income and study site.

##### Walking for specific leisure purposes

Both studies (one reporting by age group) that examined walking for exercise found that more women walked than men, except in the oldest age group (60+), in which more men walked than women (Fig. 2a and b). Similarly, both studies reporting data on walking for fun or pleasure found that more women walked for fun than men (Fig. 2a). However, both studies of dog-walking reported no significant gender difference over all ages, or in separate age groups (Fig. 2a and b).

#### Gender differences in walking for transport

Most studies looked at walking for transport to any destination, while a smaller number of studies looked at walking to particular destinations, such as work. We report results where more than one study investigated gender differences in walking to the same destination.

##### Walking for transport (general)

**Fig. 3:**
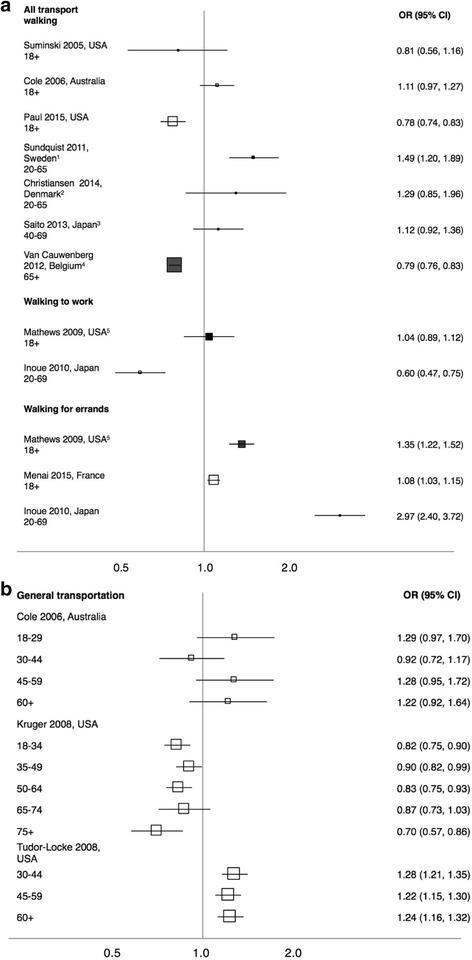
Forest plot showing odds ratios for the prevalence of walking for transport amongst women in comparison to men among (**a**) all participants and (**b**) by age group. Clear markers indicate bivariate analysis; filled markers indicate multivariate analysis, and footnotes detail control variables included in each model. Marker size is proportional to the sample size of the study. ^1^ Age, family income, marital status, neighbourhood socioeconomic status, neighbourhood walkability. ^2^ Education, income, neighbourhood walkability, life stage, distance to work or education, nativity, destination accessibility. ^3^ Age, education, employment, household economic status, presence of children in household, health, car ownership, access to shops, presence of sidewalks. ^4^ Age, education, functional limitations, area of residence. ^5^ Education and employment

There is no evidence for a consistent gender difference in participation in walking for transport (Fig. 3a). Nor, in studies examining particular age groups, is there evidence of a change in the odds ratio with age, except that in the two studies reporting on the oldest age groups (Kruger, USA, 75+ [32]; van Cauwenberg, Belgium, 65+ [49]) more men walked for transport than women (Fig. 3a and b).

Liao (Taiwan, 20-64) [34] found no difference in the proportion of men and women who walked for 150 min per week (OR 0.88 (0.58, 1.33), adjusting for age, residential area, education, occupation, marital status, living status (sic), BMI and vehicle ownership).

Van Dyck (International, 20-65) [50] found no gender difference in weekly minutes of walking for transport (OR 0.96 (0.89, 1.04)), adjusting for age, income, marital status, education, BMI, study site).

One study examined the number of walks taken for transport per week in middle-aged adults and reported no significant gender difference (mean difference = 0.01 (−0.09, 0.11)), after adjusting for car ownership, bicycle ownership, dog ownership, employment, disability, income, and neighbourhood factors (Lee, USA, 55-65) [33].

##### Walking to specific destinations

Inoue (Japan, 20-69) [29] found that fewer women walked to get to work than men, while Mathews (USA, 18+) [37] found no gender difference, controlling for education and employment (Fig. 3a).

Three studies looked at gender differences in walking for errands (Fig. 3a), all reporting that more women walked for this purpose than men. However, Oliver (Canada, 19+) [39] reported no gender difference in the proportion walking for errands for one hour or more per week (OR 0.97-1.00 (0.79-0.81, 1.19-1.24)) in five models testing for associations between different land use characteristics and walking) after controlling for age, income, marital status, and factors related to neighbourhood and health.

#### Gender differences in total walking

**Fig. 4:**
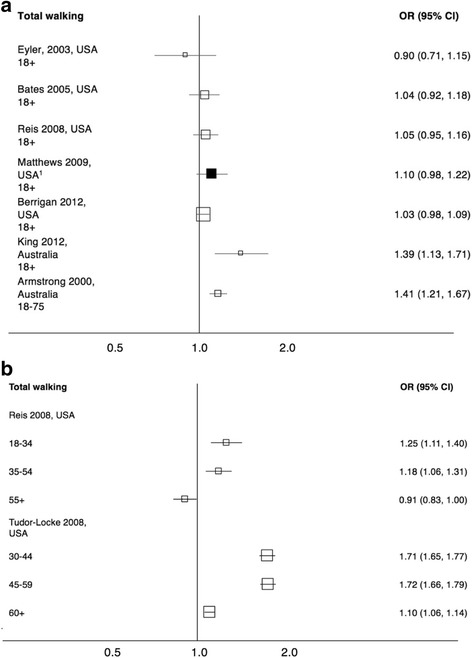
Forest plot showing odds ratios for the prevalence of total walking amongst women in comparison to men among (**a**) all participants and (**b**) by age group. Clear markers indicate bivariate analysis; filled markers indicate multivariate analysis, and footnotes detail control variables included in each model. Marker size is proportional to the sample size of the study. ^1^ Education and employment

There was no evidence for a gender difference in the prevalence of walking for any purpose in studies including all ages from the USA. Data reported by age group (in two studies from the USA, Fig. 4b) suggest that at younger ages more women walk than men, but at older ages the gender difference is very small. However, both Australian studies looking at wide age ranges reported that the prevalence of walking was higher in women than in men (Fig. 4a).

A study conducted across nearly all adult ages in the Czech Republic reported that a greater proportion of women than men walked for 150 min per week (OR 1.46 (1.34, 1.68)) (Frömel, Czech Republic, 25+) [26].

In older age groups, Hörder (Sweden, 75) [28] found no significant difference in the proportion of women and men achieving 75 min walking per week (OR 0.97 (0.63, 1.37)), whereas Satariano (US, 65+) [44] found that more men than women walked for more than 150 min per week (OR 0.56 (0.43, 0.74)).

Three studies reported on gender differences in ‘regular substantial walking’. One found that fewer women engaged in ‘regular substantial walking’ than men (OR 0.90 (0.88, 0.92)) (Ryu, South Korea, 19+) [42]. Granner (USA, 18+) [27] and Wen (USA, 18+) [52] reported non-significant odds ratios of 0.90 (0.75, 1.08) and 0.96 (0.90, 1.03) respectively (Wen adjusted for age, ethnicity, marital status, employment, education, income and weight category).

## Discussion

This review reveals that women report a higher prevalence than men of walking for leisure, for exercise, and for fun when all ages are considered together. This finding was largely consistent across countries. However, an international study found no gender difference in duration of walking for leisure in adults aged between 20 and 65. Thus it is possible that while more women than men walk for leisure, when men walk for leisure they walk further than women. There appears to be no gender difference in the prevalence of dog-walking. Our most striking finding is a consistent pattern whereby more women than men walk for leisure at young adult ages, a difference that progressively declines with age and then reverses, until in the oldest age groups (aged 60-70 and above) more men than women walked for leisure.

Walking for leisure is an activity that women can undertake with children [53] and it is possible that child-care plays a role in the relatively high levels of walking for leisure in younger women. In many high-income countries more of young and middle-aged women’s leisure time than of men’s leisure time is combined with unpaid work, usually in the form of child care, as shown by multinational time-use data from a subset of ‘Modern Western’ countries [54]. The fact that walking requires less confidence in the body’s capacity than most other forms of leisure physical activities may also encourage walking for leisure amongst women [55].

The decrease in gender difference in participation in walking for leisure across younger and middle-aged groups derives mainly from a gradual increase in participation in walking for leisure with age by men [22, 25, 31, 32, 47]. Young men’s relatively high levels of participation in sports and exercise decline with age, as reported for the UK [56] and the USA [57], and it is possible that men adopt walking for leisure as a replacement for more vigorous activities as they get older.

In the oldest age groups, the proportion of men walking for leisure declines, but the proportion of women walking for leisure declines more [22, 25, 31, 32, 47]. This pattern may reflect differences in ability to walk in older age. A British study found that “mobility limitation” rises faster with age in women than in men [58], probably because of higher levels of morbidity in older women than in older men, including musculoskeletal problems [59–61]. It is also plausible that increased physical limitations exacerbate gendered concerns about personal safety, which appear to constrain women’s walking [62]; older women with limited mobility may feel particularly vulnerable in public spaces. On the other hand, it is also possible that a reluctance to walk contributes to a decline in functional mobility in older women and further research is needed to disentangle the causes of the gender difference in the decline in walking for leisure in older age.

There was no evidence for a consistent gender difference in participation in walking for transport and no gender difference in the duration or the frequency of walking for transport was observed. However, there was some evidence that the purpose of men’s and women’s walking for transport may differ. In the one study (from Japan) that reported gender differences in walking to work without controlling for employment status, more men than women walked to work [29]. This raises the possibility that more men walk to work than women because more men are in employment [63], although such an effect would vary across countries since gender differences in labour force participation are variable (and relatively large in Japan).

More women than men walked for errands, in line with a general trend for women to devote more time and make more trips than men to serve their household. For example, time-use data collected in 1995 in the USA found that in European American dual-earner married couples both part-time and full-time employed women spent significantly more time per day than employed men serving household needs outside the home [64]. Data from the USA in the 2000s show that women in couples with children made 2.0 trips to serve children’s needs for every 1 trip made by a man, and 1.7 trips to buy groceries for every 1 trip by a man [65]. In Germany, while the ratio of trips made for shopping by women compared to men fell sharply between 1976 and 2008, it was still 1.28 in 2008 [14]. In this context, it is not surprising that more women than men report walking for the purpose of running errands.

There is no gender difference in participation in total walking in the USA when all ages are considered together. There is some evidence of an age-related pattern whereby at younger ages more women walk than men, but at older ages this gender difference is much smaller, or may reverse (consistent with patterns for walking for leisure and with the results for walking for transport in the oldest age groups). As for walking for leisure, this change in the gender difference with age appears to arise because the proportion of women who walk declines faster with age than the proportion of men [41, 47]. There was also evidence that in Australia more women walk than men. A tentative suggestion, therefore, is that, at least at younger ages, more women than men walk (in total), but that there appears to be geographical variation in gender differences in total walking in middle and older age groups.

This review has some limitations. All of the data were self-reported and while some studies used validated instruments such as the IPAQ, others used similar methods of assessment without specific validation. It is possible that the self-reports of men and women in relation to walking are subject to different biases, which would affect the validity of our findings. Most studies reported on the proportion of men and women who walked during a given period of time. There was limited information on the frequency or duration of walking, which would allow more insight into walking patterns. All the data were collected cross-sectionally and patterns of changing differences over the life-course may therefore reflect cohort effects as well as aging effects. The fact that relevant data were usually reported incidentally in papers focused on other topics means that some eligible studies may not have been retrieved by the search strategy. We suggest there is relatively little risk of publication bias having affected the availability of data for this review, largely because gender was not the main focus of the vast majority of included studies.

## Conclusions

The main contribution of this review is to reveal and draw attention to previously largely unrecognised but apparently consistent gender differences in walking for different purposes amongst adults, principally higher levels of walking for leisure and walking for errands by women. We also identified a clear age-related pattern in gender differences in walking for leisure, whereby at younger ages more women than men walk for leisure, a difference that gradually declines and then reverses, so that in the oldest age groups, more men walk for leisure than women.

We have offered some possible explanations for these findings, noting the strongly gendered nature of daily life as reflected in time-use studies, labour force participation statistics and in surveys of general mobility. It seems plausible that women’s childcare responsibilities, perceptions of risk and functional limitations in older life affect their opportunities for, and experiences of, walking. However, the gendered patterns of walking revealed here suggest that more research is needed to improve our understanding of how walking fits into the lives of women and men across the life-course. Particular attention should be paid to investigating the causes of gender differences in the impact of aging on walking, with a view to designing successful and possibly gender-differentiated interventions to maintain walking levels into older age. Maintaining physical activity in older age is important for health and wellbeing and walking has great potential to help older adults achieve recommended levels of physical activity [66].We suggest that approaches to understanding walking as a social practice, embedded in, and shaped by, complex social worlds [67, 68], in which gender plays a powerful role, are likely to be particularly helpful.

## Additional file

Additional file 1: Study setting, sample size, recruitment strategy, participant characteristics and outcome measures of included studies. (DOCX 25 kb)

## Abbreviations

TRID: Transportation research international documentation
CASP: Critical appraisal skills programme
IPAQ: International physical activity questionnaire
OR: Odds ratio
