# Supplementary material for: Gender differences in walking (for leisure, transport and in total) across adult life: a systematic review
Source: BMC Public Health. 2017 Apr 20;17:341. doi: 10.1186/s12889-017-4253-4 (PMC5397769; doi:10.1186/s12889-017-4253-4)
Supplement: Additional file 1: — Study setting, sample size, recruitment strategy, participant characteristics and outcome measures of included studies. (DOCX 25 kb) [file 12889_2017_4253_MOESM1_ESM.docx]

| Study | Study Setting and Name (if applicable) | Sample Size (% women) | Recruitment Strategy | Age and Key Characteristics of Participants | Outcome Measures included in Review |
| --- | --- | --- | --- | --- | --- |
| Armstrong et al. 2000 | Australia, Nationwide *National Physical Activity Survey* | 3841 (50%) | Random selection of individuals within households randomly selected from telephone directory | 18-75 | Walked ≥10 minutes in past week |
| Bates et al. 2005 | USA, Nationwide *National Physical Activity Survey* | 6626 (59%) | Random selection of individuals within households selected by random-digit dialling | 18+ | Walked ≥10 minutes in usual week |
| Beenackers et al. 2013 | Netherlands, Eindhoven *Dutch GLOBE study* | 4395 (53%) | Sample from population register stratified by age, degree of urbanization, and socioeconomic position | 25-75, Dutch nationality | Walked during leisure-time in usual week |
| Berrigan et al. 2012 | USA, Nationwide *NHIS* | 26328 (55%) 23129 (55%) | Multistage probability design to provide representative sampling of households and non-institutional group quarters | 18+ | Walked ≥10 minutes in past 7 days |
| Christiansen et al. 2014 | Denmark, Aarhus *IPEN Study* | 642 (56%) | Random selection from districts chosen to represent combinations of high- or low-walkability and high- or low-income | 20-65 | Walked for transport ≥10 minutes in past week |
| Cole et al. 2006 | Australia, New South Wales *Physical Activity Survey for New South Wales* | 3392 (51%) | Random selection of individuals within households randomly selected from telephone directory | 18+ | Walked for recreation in past 2 weeks  Walked for transport in past 2 weeks |
| Duncan & Mummery 2005 | Australia, Queensland | 741 (51.5%) | Random using a two-stage stratified sampling design | 18+ | Walked for recreation ≥10 minutes in past 7 days |
| Eyler et al. 2003 | USA, Nationwide | 1818 (67%) | Random-digit-dialling across the US, oversampling low-income zip codes | 18+ | Walked ≥10 minutes at a time in a usual week |
| Foster et al. 2009 | UK, Norwich *EPIC-Norfolk study* | 13927 (56%) | Through general practice lists, except those deemed unsuitable by general practitioner | 48-77 | Walked for recreation in past year |
| Frömel et al. 2009 | Czech Republic, Nationwide | 9950 (51%) | Random sampling (residents of every 10th residence on each block in selected locations from geocoded database) in all regions of Czech republic | 25+ | Walked ≥150 minutes over last 7 days |
| Granner et al. 2007 | USA, South Carolina | 2025 (60%) | Random selection of individuals within households selected by random digit-dialling | 18+ | Walked ≥5 times per week, ≥ 30 minutes per day |
| Hörder et al. 2013 | Sweden, Gothenberg | 637 (67%) | Individuals born in 1930 and living in Gothenberg systematically selected from the Swedish Population Register | 75 years old, no dementia | Walked ≥75 minutes per week |
| Inoue et al. 2010 | Japan, Four cities (Koganei, Tsukuba, Shizuoka, Kagoshima) | 1461 (55%) | Randomly sampled addresses, then stratified by sex, age and city of residence | 20-69 | Walked ≥ 5 minutes for leisure in past week  Walked ≥ 5 minutes for errands in past week  Walked ≥ 5 minutes to work in past week |
| King et al. 2012 | Australia, Melbourne *VicLANES study* | 2349 (56%) | Random selection from electoral roll, stratified across census districts | 18+ | Walked ≥10 minutes in past month |
| Kramer et al. 2013 | Netherlands, Nationwide  *Dutch National Health Survey* | 20046 (53%) | Random nationwide sample drawn from national population registry | 18+ | Walked for 30 minutes per week in typical week |
| Kruger et al. 2008 | USA, Nationwide *NHIS* | 29479 (56%) | Multi-stage sampling design to obtain a representative sample of adults aged 18 or older | 18+ | Walked ≥10 minutes for leisure in past week  Walked ≥10 minutes for transport in past week |
| Lee et al. 2013 | USA, Boston | 933 (59%) | Random sample of addresses with residents aged 55 or older | 55-65 | Number of transportation walking trips over last 7 days |
| Liao et al. 2015 | Taiwan, Nationwide | 1065 (49%) | Random digit telephone survey with stratified and clustered sampling design | 20-64 | Walked ≥150 minutes per week |
| Livingstone et al. 2001 | Ireland, Nationwide *North/South Ireland Food Consumption Survey* | 1379 (51%) | Random selection using the electoral register to randomly select electoral divisions then randomly select individuals within areas | 18-64, not pregnant or breastfeeding | Walked for pleasure/fun in past week |
| Martin et al. 2014 | UK, Nationwide *Medical Research Council National Survey of Health and Development Study* | 2188 (52%) | Representative sample of people born in England, Scotland, and Wales in March 1946 | 60-64 | Walked for pleasure at least weekly ≥30 minutes per episode |
| Mathews et al. 2009 | USA, California | 12036 (57%) | Random digit-dial telephone survey sampling twelve regions in California | 18+ | Walked in typical week  Walked to work in typical week  Walked to run errands in a typical week |
| Menai et al. 2015 | France, Nationwide *NutriNet-Santé Study* | 39295 (77%) | Via a multi-media campaign and direct information to members of another cohort. | 18+ | Walked ≥30 minutes for leisure per week over past 4 weeks  Walked ≥30 minutes for errands per week over past 4 weeks |
| Oliver et al. 2011 | Canada, Vancouver | 1602 (62%) | Random digit dialling within selected census blocks. | 19+ | Walked ≥ one hour per typical week to do errands  Walked ≥15 minutes per day for leisure in typical week |
| Paul et al. 2015 | USA, Nationwide *NHIS* | 24017 (55%) | Multistage probability design to provide representative sampling of households and non-institutional group quarters | 18+, able to walk | Walked ≥10 minutes for transport in past 7 days  Walked≥10 minutes for leisure in past 7 days |
| Reis et al. 2008 | USA, Nationwide *National Physical Activity and Weight Loss Survey* | 10461 (58%) | Random sample of civilian, non-institutionalised adults, oversampling non-Hispanic Black and Hispanic adults | 18+ | Walked ≥10 minutes in usual week |
| Ryu et al. 2014 | South Korea, Nationwide *Korean Community Health Survey* | 199400 (53%) | Multi-stage sampling design to obtain a representative sample | 19+ | Walked ≥30 minutes on ≥5 days a week |
| Saito et al. 2013 | Japan, Fujisawa | 2449 (51%) | Recipients of National Health Insurance, randomly selected using postal pin code and stratification by sex. | 40-69 | Walked ≥10 minutes for leisure in a typical week  Walked ≥10 minutes for transport in a typical week |
| Satariano et al. 2010 | USA, Four counties in four states (CA, IL, PA, NC) | 884 (77%) | Through senior organizations that were selected to represent quintiles of housing density | 65+, able to walk | Walked ≥150 minutes in typical week |
| Suminski et al. 2005 | USA, Midwest | 474 (56%) | Random sample of addresses | 18+, not limited in ability to be physically active due to a health condition | Walked for exercise in past 7 days  Walked a dog in past 7 days  Walked for transport in past 7 days |
| Sundquist et al. 2011 | Sweden, Stockholm *The Swedish Neighborhood and Physical Activity Study* | 2269 (55%) | Random sample from neighbourhoods selected to include high and low income and high and low walkability areas | 20-65, no serious impaired ability to walk | Walked ≥10 minutes for leisure in past 7 days  Walked ≥10 minutes for transport in past 7 days |
| Tudor-Locke & Ham 2008 | USA, Nationwide *American Time Use Survey* | 47731 (56%) | A stratified, random subsample drawn from households that completed the Current Population Survey, with oversampling of households with Hispanic or non-Hispanic black members and households with children | 15+ (but data are reported for older age groups separately) | Walked on previous day  Walking for exercise on previous day  Walked dog on previous day  Walked for transportation on previous day |
| Van Cauwenberg et al. 2012 | Belgium, Flemish municipalities *Belgian Aging Studies* | 48879 (56%) | Stratified (for age and gender) cluster random sampling of those aged over 60 | 65+ for this analysis | Walked ≥10 minutes daily for transportation |
| Van Cauwenberg et al. 2015 | Australia, Victoria *The WELL project* | 2700 (53%) | Stratified cluster random sampling | 55-65, not unable to perform physical activity due to health problems | Walked ≥10 minutes for leisure in past 7 days |
| Van Dyck et al. 2012 | International: USA (Seattle, Baltimore), Belgium (Ghent), Australia (Adelaide) *NQLS, BEPAS, and PLACE studies* | 6014 (56%) | Participants randomly selected from high and low walkable and high and low income neighbourhoods. | 20-65 | Minutes of transport-related walking over past 7 days |
| Van Dyck et al. 2013 | International: USA (Seattle, Baltimore), Belgium (Ghent), Australia (Adelaide) *NQLS, BEPAS, and PLACE studies* | 6014 (56%) | Participants randomly selected from high and low walkable and high and low income neighbourhoods. | 20-65 | Minutes of recreational walking over past 7 days |
| Wen et al. 2007 | USA, California *California Health Interview Survey* | 41545 (51%) | Random-digit-dialling | 18+ | Walked ≥30 minutes on ≥5 days a week |
